# The two-component system expression patterns and immune regulatory mechanism of *Vibrio parahaemolyticus* with different genotypes at the early stage of infection in THP-1 cells

**DOI:** 10.1128/msystems.00237-23

**Published:** 2023-07-11

**Authors:** Yuan-Yuan Meng, Jun-Hui Peng, Jiang Qian, Fu-Lin Fei, Ying-Ying Guo, Ying-Jie Pan, Yong Zhao, Hai-Quan Liu

**Affiliations:** 1 College of Food Science and Technology, Shanghai Ocean University, Shanghai, China; 2 Shanghai Fisheries Research Institute, Shanghai Fisheries Technical Extension Station, Shanghai, China; 3 Laboratory of Quality & Safety Risk Assessment for Aquatic Products on Storage and Preservation, Ministry of Agriculture and Rural Affairs, Shanghai, China; 4 Shanghai Engineering Research Center of Aquatic-Product Processing & Preservation, Shanghai, China; University of California Davis, Sacramento, California, USA

**Keywords:** *Vibrio parahaemolyticus*, two-component system, expression pattern, interaction, innate immune

## Abstract

*Vibrio parahaemolyticus* must endure various challenging circumstances while being swallowed by phagocytes of the innate immune system. Moreover, bacteria should recognize and react to environmental signals quickly in host cells. Two-component system (TCS) is an important way for bacteria to perceive external environmental signals and transmit them to the interior to trigger the associated regulatory mechanism. However, the regulatory function of *V. parahaemolyticus* TCS in innate immune cells is unclear. Here, the expression patterns of TCS in *V. parahaemolyticus*-infected THP-1 cell-derived macrophages at the early stage were studied for the first time. Based on protein-protein interaction network analysis, we mined and analyzed seven critical TCS genes with excellent research value in the *V. parahaemolyticus* regulating macrophages, as shown below. VP1503, VP1502, VPA0021, and VPA0182 could regulate the ATP-binding-cassette (ABC) transport system. VP1735, uvrY, and peuR might interact with thermostable hemolysin proteins, DNA cleavage-related proteins, and TonB-dependent siderophore enterobactin receptor, respectively, which may assist *V. parahaemolyticus* in infected macrophages. Subsequently, the potential immune escape pathways of *V. parahaemolyticus* regulating macrophages were explored by RNA-seq. The results showed that *V. parahaemolyticus* might infect macrophages by controlling apoptosis, actin cytoskeleton, and cytokines. In addition, we found that the TCS (peuS/R) could enhance the toxicity of *V. parahaemolyticus* to macrophages and might contribute to the activation of macrophage apoptosis.

**IMPORTANCE:**

This study could offer crucial new insights into the pathogenicity of *V. parahaemolyticus* without *tdh* and *trh* genes. In addition, we also provided a novel direction of inquiry into the pathogenic mechanism of *V. parahaemolyticus* and suggested several TCS key genes that may assist *V. parahaemolyticus* in innate immune regulation and interaction.

## INTRODUCTION

*Vibrio parahaemolyticus* is a foodborne pathogen with a multi-host range that can infect marine animals and humans (1). The most widely studied virulence factors of *V. parahaemolyticus* have thermostable hemolysin (*tdh/trh*) and Type III secretory system (T3SS1 and T3SS2) (2 - 4). It is generally agreed upon that possessing more virulence factors will have stronger pathogenicity. However, among the 42 samples of *V. parahaemolyticus* with different genotypes that we isolated from fecal samples of patients treated in Shanghai hospital, most of the pathogens carried just one thermostable hemolysin (*tdh* or *trh*), a small number of pathogens had both *tdh* and *trh*, and there is also a pathogen without *tdh* and *trh* (5). Furthermore, VopZ, a T3SS2 effector protein, was discovered to be important for intestinal colonization and diarrheagenic, which is essential for the pathogenicity of *V. parahaemolyticus* (6). However, we found that it was not detectable in some strains (7). Zhang et al. found that VopC was critical for *V. parahaemolyticus* T3SS2-mediated invasion (8), but another research reveals that VopC is not necessary for pathogenicity in an animal infection model (9). And several strains of 42 samples were undetectable VopC (5). Since we cannot ignore the diversity of pathogenic *V. parahaemolyticus* in clinical practice, we chose *V. parahaemolyticus* of different genotypes as research objects to investigate new pathogenic factors.

Generally, *V. parahaemolyticus* infects humans through the skin, gastrointestinal wounds, and diet. It must overcome several challenges to cause illness, and the first hurdle is the innate immune system (10). When the immune phagocytes ingest the bacteria, they will be under various extreme conditions, including an acidic pH, hypoxia, reactive oxygen species, reactive nitrogen species, and nutritional deprivation (11). Therefore, recognizing and reacting quickly to external environmental signals is crucial for bacterial survival in the cell. Two-component system (TCS) is a critical process for bacteria to sense environmental signals and transmit them to the interiors to activate regulatory mechanisms (12). It is known that TCS is composed of a transmembrane sensor protein known as histidine kinase (HK) on the bacterial membrane and a response regulator (RR) in the cytoplasm (12). According to the P2CS (http://www.p2cs.org) prokaryote database, *V. parahaemolyticus* had 50 HKs and 55 RRs. The HK transfers its phosphate group to RR, and RR changes downstream target gene expression to control intercellular communication, environmental adaptation, growth, adhesion, and other processes. Moreover, TCS can also influence bacterial pathogenicity by regulating itself or interfering with the host immune system (13, 14). For example, *Salmonella typhimurium* recognizes the host by a surface-located HK PhoP sensor and phosphorylates the intracellular effector PhoQ, thus promoting the bacterial expression of lipid A-modifying enzymes. Modification of lipid A attenuates toll-like receptor 4 (TLR4)-mediated nuclear factor kappa-B (NF-κB), thereby inhibiting the generation of inflammatory cytokines and preventing the immune system from responding normally (15).

Studies have shown that *V. parahaemolyticus* effector proteins, VopQ and VopS*,* can inhibit the activation of the nucleotide-binding oligomerization domain (NOD), leucine-rich repeat and caspase recruitment domain-containing 4 (NLRC4) by inducing autophagy and cell division cycle 42 (Cdc42) inactivation in macrophages, respectively (2). And VopQ can also prevent phagocytosis (16). However, it is fully unclear how *V. parahaemolyticus* interacts with human innate immune cells. The RNA-seq has greatly helped researchers fully understand pathogenic mechanisms and key pathogenic factors of pathogens. Nevertheless, persistent infection worsens intracellular pathogen status, particularly non-parasitic pathogens, which is difficult for RNA-seq technology with strict requirements for sample RNA. Moreover, the content of pathogens in the cell is too small, resulting in a low proportion of pathogens in the total RNA extracted, which also increases difficulty to fully comprehend the pathogenic mechanisms of bacteria in the interaction with host immune cells (17). Therefore, in this study, we chose the early infection stage of immune cells and utilized quantitative real-time PCR (qPCR) to examine the transcriptional expression levels of TCS in *V. parahaemolyticus*. qPCR is a reliable and rapid method to assess the level of target gene expression, which is particularly sensitive to the detection of some low-copy mRNAs (18). This method aids in understanding the changes of TCS in *V. parahaemolyticus*-infected immune cells. In addition, we chose THP-1 cell-derived macrophages as an innate immune cell model because numerous studies have demonstrated that THP-1 cells are a suitable model for early infection (19).

This study aimed to identify the expression patterns of TCS in *V. parahaemolyticus* with various genotypes under macrophage stress and to explore the potential critical genes and mechanisms of *V. parahaemolyticus* regulating host innate immunity. First, we determined that the optimal time for *V. parahaemolyticus* to infect cells was 3.5 h (infection for 1.5 h and gentamicin treatment for 2 h) based on intracellular survival bacterial counts. And the TCS expression profiles of *V. parahaemolyticus* were detected by quantitative real-time PCR (qRT-PCR). On this basis, differently expressed TCS genes (DETGs) and their interacted proteins were analyzed by protein-protein interaction (PPI) network analysis. Subsequently, the RNA-seq was used to comprehensively understand the changes at the transcription level of macrophages at 3.5 h after *V. parahaemolyticus* infection. Furthermore, we explored the potential role of TCS in regulating macrophages by the knockout of critical TCS genes.

In this study, the interaction of *V. parahaemolyticus* TCS in regulating innate immune cells was studied for the first time. Moreover, the object of the study was comprehensive, including *V. parahaemolyticus* of various genotypes. This study is beneficial to a thorough understanding of the potential function of TCS during host cell infection and provides new directions for studying *V. parahaemolyticus* in innate immune regulation.

## RESULTS

### Intracellular survival of *V. parahaemolyticus*

**Fig 1:**
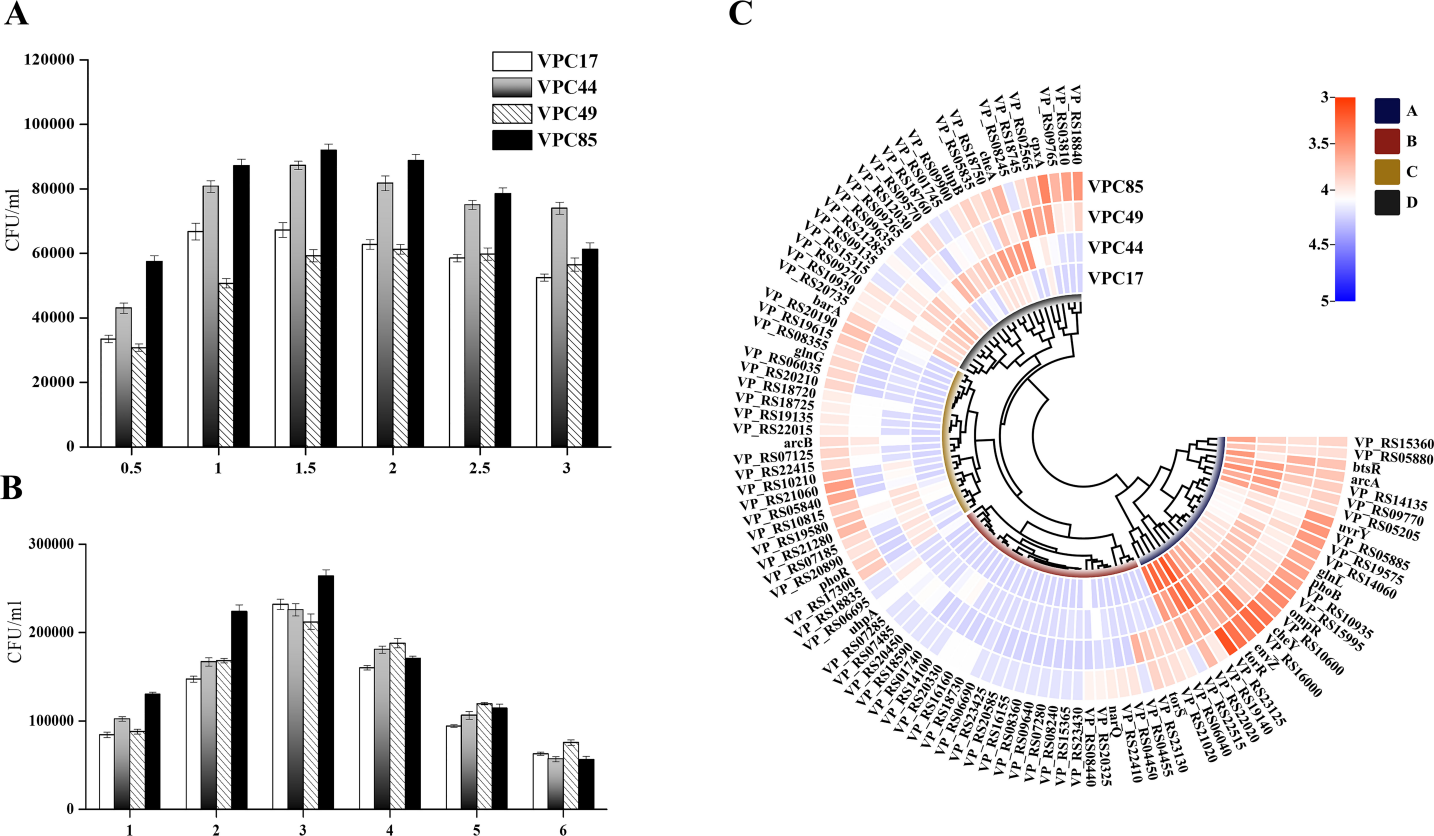
Intracellular survival and TCS expression patterns of *V. parahaemolyticus*-infected THP-1 cell-derived macrophages. (**A**) The macrophages were infected with *V. parahaemolyticus*. Results are calculated from intracellular *V. parahaemolyticus* collected at 0–3 h. (**B**) The macrophages were infected with *V. parahaemolyticus* for 2 h and treated with the gentamicin-containing medium for 6 h. The amount of intracellular *V. parahaemolyticus* was determined at 0–6 h. (**C**) Expression pattern of TCS in *V. parahaemolyticus*-infected macrophages. The macrophages infected with *V. parahaemolyticus* for 1.5 h and treated with the gentamicin-containing medium for 2 h. Results were plotted by the expression level of the TCS genes in intracellular *V. parahaemolyticus* after infection.

To evaluate the number of *V. parahaemolyticus* in macrophages, *V. parahaemolyticus* with four genotypes [VPC17 (*tdh*+/*trh*−), VPC44 (*tdh*−/*trh*−), VPC49 (*tdh+*/*trh*+), and VPC85 (*tdh*−/*trh*+)] was used to infect cells in the antibiotic-free medium at 0–3 h. The results showed that the number of *V. parahaemolyticus* increased within 1.5 h but diminished after more than 2 h (Fig. 1A). During infection, not all bacteria were in direct touch with cells, and extracellular *V. parahaemolyticus* proliferated quickly in the antibiotic-free environment. The drop in intracellular bacteria may be due to a considerable increase in external bacteria, intensifying the attack on cells and continuing cell lysis.

We examined that four strains could not grow in the medium containing 100 ng/mL gentamicin. Then, the cells were infected with four strains for 1.5 h; the extracellular bacteria were removed and treated with the gentamicin-containing medium for 0–6 h. The results showed that the cell’s bacterial population peaked at 3 h and decreased between 4 h and 5 h (Fig. 1B). This may be due to the proliferation of intracellular bacteria leading to cell lysis overflow (8). The number of VPC85 was much lower at 4–6 h than the other three strains, showing that the intracellular bacteria proliferated rapidly and aggravated macrophage lysis. Based on the above research results, we determined the *V. parahaemolyticus* infection time to be 1.5 h and the gentamicin treatment time to be 2 h.

### Expression patterns of TCS in *V. parahaemolyticus*-infected macrophages

The expression patterns of TCS in *V. parahaemolyticus* with four genotypes were shown by cluster heatmap (Fig. 1C). The red square in the heatmap indicates high expression, and the blue one indicates low expression. After infection with macrophages, 63, 40, 43, and 32 TCS genes could not be detected in VPC17, VPC44, VPC49, and VPC85, respectively. These *V. parahaemolyticus* TCS genes did not express or had extremely low expression abundance, suggesting that most TCS genes had changed considerably under macrophage stress. The expression patterns of TCS were separated into four categories; groups A and B were similar. Moreover, VPC85 in group C differed most from the other three strains. The common genes among the four strains were located in groups A and B, indicating that this part of TCS genes may contain essential genes that function as regulators when *V. parahaemolyticus* infects macrophages.

### TCS in *V. parahaemolyticus* before and after infected macrophages

**Fig 2:**
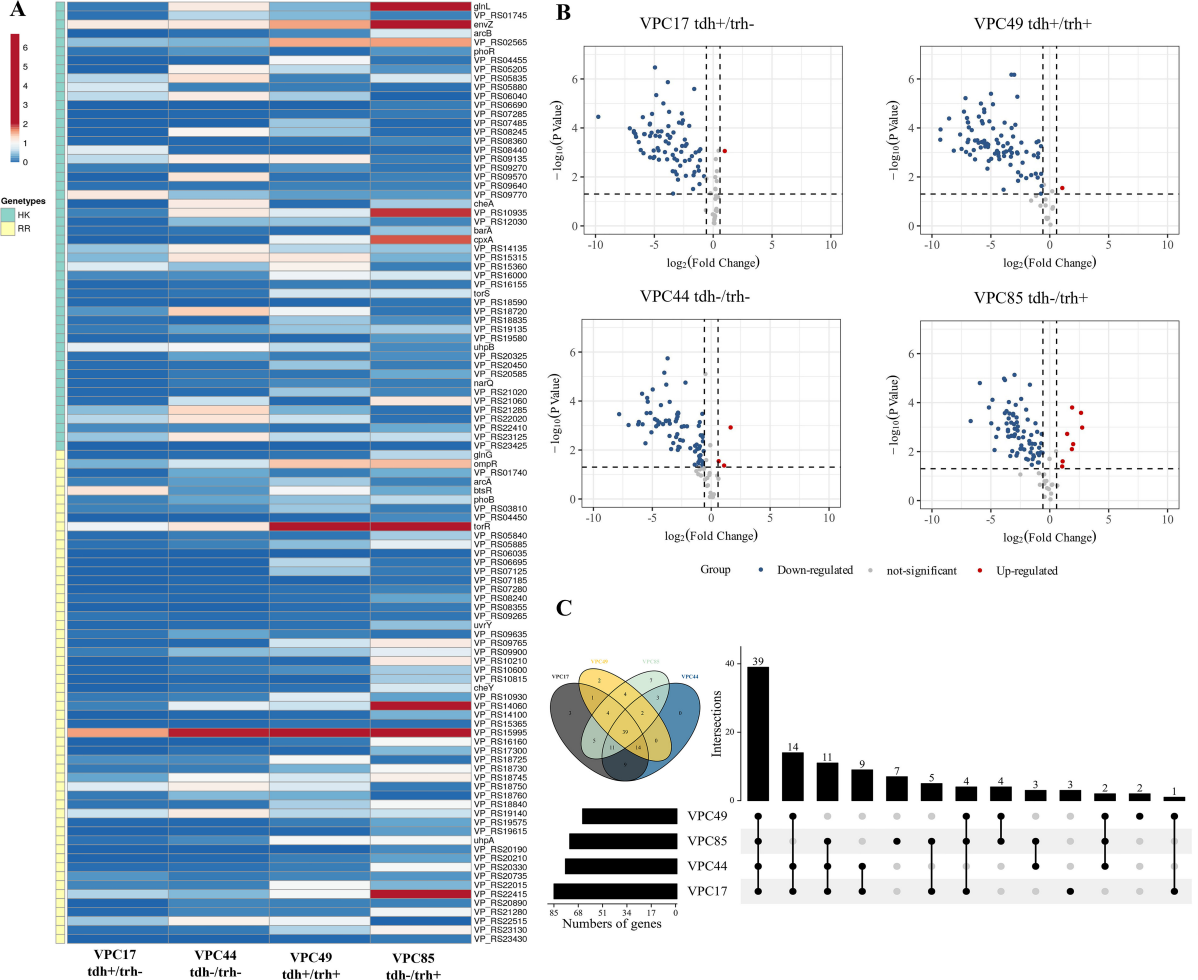
TCS in *V. parahaemolyticus* before and after infection. (**A**) The expression profiles of TCS in VPC17, VPC44, VPC49, and VPC85 before and after infection. Results were obtained by comparing the experimental group with the control group. The expression level of the TCS genes in uninfected strains was used as the control group, and the expression level of the TCS genes in intracellular strains after infection was used as the experimental group. (**B**) The volcano plot of DETGs in VPC17, VPC44, VPC49, and VPC85. According to the expression level of the TCS genes in four strains before and after infection. The screening conditions for DETGs were fold change >1.5 and *P* value < 0.05. (**C**) The Venn diagram and upset of DETGs in VPC17, VPC44, VPC49, and VPC85. The results were the number of DETGs with similar expression patterns in four strains.

We compared expressed levels of *V. parahaemolyticus* TCS genes between uninfected and infected (Fig. 2A). The red part in the map indicates up-regulated genes, and the blue one indicates down-regulated genes. In VPC17, VPC44, VPC49, and VPC85, the number of genes with fold change >1 was 4, 20, 8, and 15, respectively (*P* < 0.05), and the number of genes with fold change <1 was 100, 83, 91, and 85, respectively (*P* < 0.05). We selected fold change >1.5, <0.5, and *P* < 0.05 as DETGs, of which 1, 1, 3, and 8 genes were significantly up-regulated, and 84, 77, 60, and 66 genes were significantly down-regulated (Fig. 2B). We found that most TCS genes of strains were down-regulated, indicating that TCS was inhibited under macrophage stress. Notably, a few genes were still up-regulated in all four strains, such as VP_ RS15995 (VPA0148). According to the Venn diagram, there were 39 identical DETGs in the four strains (*P* < 0.05) (Fig. 2C). And 21 of the 39 DETGs had similar expression patterns combined with the expression pattern of TCS after infection. We selected these 21 DETGs as potential regulatory genes for the study of *V. parahaemolyticus* regulating macrophages; the information and Kyoto Encyclopedia of Genes and Genomes (KEGG) description of these 21 DETGs were shown in Table 1.

**TABLE 1:** The details of 21 DETGs

Gene	Old locus tag	Functional domain	KEGG description
VP_RS06690	VP1375	Histidine kinase, classic, 1 cNMP_binding, 1 HisKA_MA, 1 HATPase_c	–^*a*^
VP_RS07285	VP1503	Histidine kinase, classic 1 HAMP, 1 HisKA, 1 HATPase_c	NtrC family
VP_RS08360	VP1735	Histidine kinase, classic 1 HAMP, 1 HisKA, 1 HATPase_c	OmpR family, qseC, flagella regulon
VP_RS09640	VP1984	Histidine kinase, classic 1 HisKA, 1 HATPase_c	–
VP_RS16155	VPA0182	Histidine kinase, classic 1 PAS, 1 HisKA, 1 HATPase_c	Putative C4-dicarboxylate transport sensor protein
VP_RS18590	VPA0710	Histidine kinase, hybrid 1 HisKA_MA, 1 HATPase_c, 1 Response_reg,	CAI-1 autoinducer sensor kinase/phosphatase cqsS, luciferase operon
VP_RS20450	VPA1100	Histidine kinase, unorthodox 1 HisKA, 1 HATPase_c, 1 Response_reg, 1 Hpt	–
VP_RS20585	VPA1130	Histidine kinase, hybrid 1 HAMP, 1 PAS_4, 1 HisKA, 1 HATPase_c, 1 Response_reg	–
narQ	VPA1196	Histidine kinase, classic 1 HAMP, 1 HisKA_3, 1 HATPase_c	NarL family, narQ, nitrogen reductase, formate dehydrogenase, fumarate reductase
VP_RS22410	VPA1515	Histidine kinase, classic 1 HisKA, 1 HATPase_c	–
VP_RS23425	VPA1731	Histidine kinase, classic 1 HisKA, 1 HATPase_c	NtrC family, dctB, C4-dicarboxylate transport sensor histidine kinase
VP_RS01740	VP0361	Response regulator, OmpR family 1 Response_reg, 1 Trans_reg_C	OmpR family, cusR, copper resistance phosphate regulon response regulator
VP_RS04450	VP0914	Response regulator, NtrC family 1 Response_reg, 1 AAA_5, 1 HTH_8	NtrC family, dctD, C4-dicarboxylate transport response regulator
VP_RS07280	VP1502	Response regulator, NtrC family 1 Response_reg, 1 AAA_5, 1 HTH_8	Sigma-54-dependent transcriptional regulator
VP_RS08240	VP1711	Response regulator, Response_reg, 1 CitT	citB family
uvrY	VP1945	Response regulator, NarL family 1 Response_reg, 1 GerE	NarL family, uvrY, invasion response regulator and carbon storage regulator
VP_RS14100	VP2866	Response regulator, NarL family 1 Response_reg, 1 HTH_LUXR	LuxR family
VP_RS15365	VPA0021	Response regulator, LytTR family 1 Response_reg, 1 LytTR	–
VP_RS15995	VPA0148	Response regulator, OmpR family 1 Response_reg, 1 Trans_reg_C	–
VP_RS19575	VPA0919	Response regulator, OmpR family 1 Response_reg, 1 Trans_reg_C	–
VP_RS23430	VPA1732	Response regulator, PrrA family 1 Response_reg, 1 HTH_8	–

^*a*^ "–" means unknown.

### PPI analysis of DETGs

**Fig 3:**
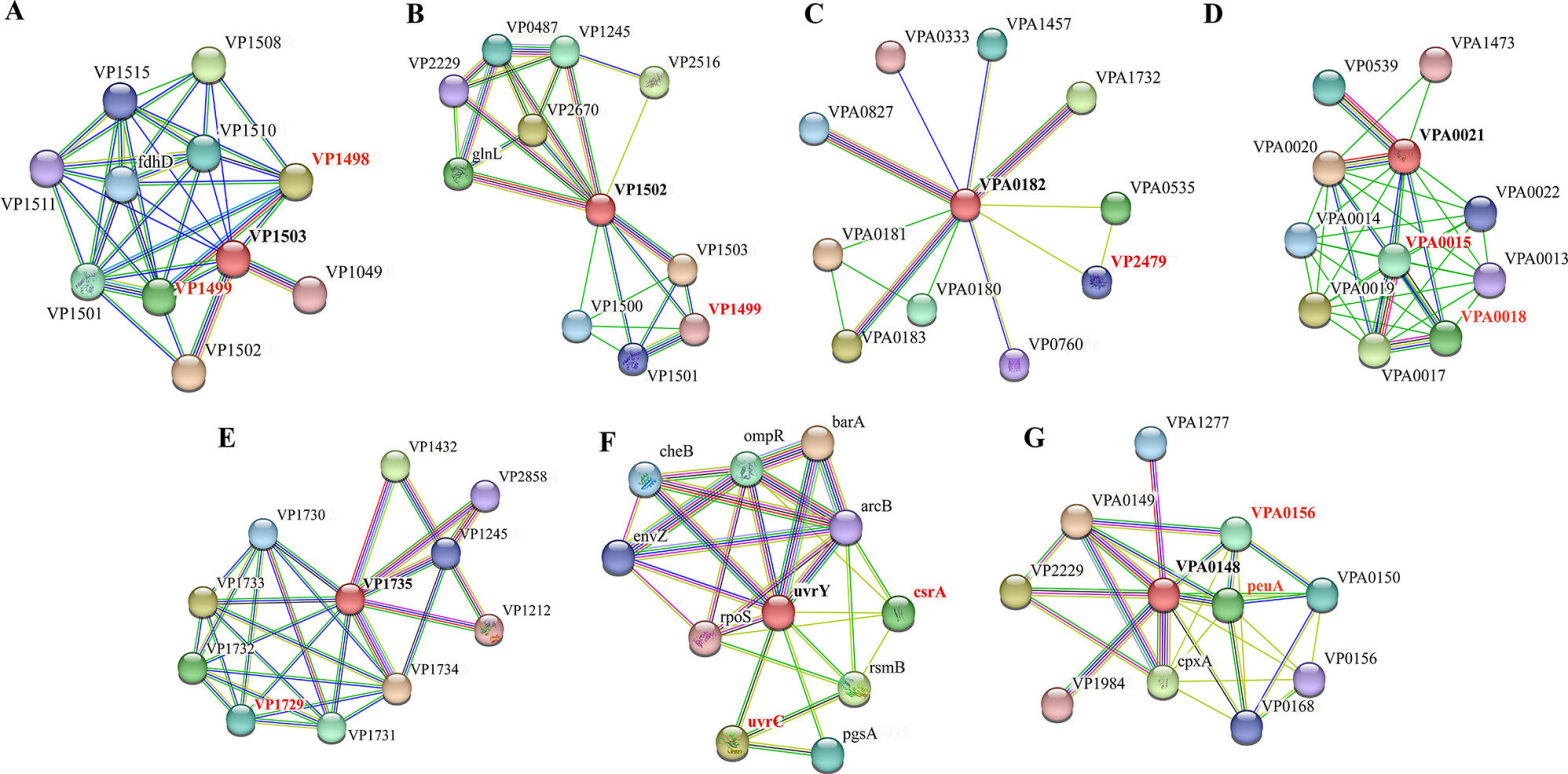
PPI network analysis of DETGs.

To explore the potential regulatory targets of 21 DETGs in *V. parahaemolyticus*, we analyzed their interacting proteins by String. Notable DETGs were VP_RS07285 (VP1503), VP_RS07280 (VP1502), VP_RS16155 (VPA0182), VP_RS15365 (VPA0021), VP_RS08360 (VP1735), VP_RS01740 (VP0361), uvrY (VP1945), and VP_ RS15995 (VPA0148). The PPI analysis revealed that VP1503, VP1502, VPA0182, and VPA0021 might interact with ABC transporter proteins. VP1503 protein was predicted to interact with ABC transporter permease encoded by VP1499 and ATP-binding protein encoded by VP1498 (Fig. 3A). VP1502 protein may interact with VP1499 protein (Fig. 3B). VPA0182 protein was predicted to interact with ABC transporter substrate-binding protein encoded by VP2479 (Fig. 3C). VPA0021 protein may interact with VPA0015-encoded ATP-binding protein (Fig. 3D). The ABC protein family plays various roles in the intake of nutrients, metabolites, excretion of bacterial toxic compounds, biofilm formation, invasion, and host colonization (20). The results suggested that regulating ABC transporters by TCS may have research value for the mechanisms of *V. parahaemolyticus* in regulating macrophages.

VPA0021 can also interact with VPA0018-encoded outer membrane lipoprotein (Fig. 3D). VPA0018 protein contains a Lol A domain that moves lipoprotein from the inner membrane to the outer membrane. Then, the lipoprotein is received and located inside the cell’s outer membrane through the membrane receptor protein Lol B. This process plays a vital role in assembling bacterial outer membrane structure and regulating bacterial life activities (21).

Figure 3E shows that the VP1735 protein might interact with the thermostable hemolysin protein encoded by VP1729. Thermostable hemolysin protein has hemolytic activity, enterotoxin activity, cardiotoxicity, and cytotoxicity (22 - 25). In addition, VP1735 may also interact with the iron-containing redox enzyme family protein (VP1731-encoded), which may play a role in iron uptake.

The uvrC is a noteworthy protein in the proteins interacting with uvrY (Fig. 3F). The uvrC contains the GIY-YIG domain, which aids in repairing DNA damage and preserving genomic stability (26). In addition, uvrY can also interact with csrA (Fig. 3F). The post-transcriptional regulator csrA plays a central role in adapting pathogens to infect animal hosts (27, 28).

VPA0148 might interact with the protein encoded by VPA0156 that contains the tetratricopeptide repeat (TPR) domain. Proteins containing TPR can mediate PPIs, assemble multiprotein complexes, and participate in several biological activities, including regulating the cell cycle, transcriptional control, and transporting proteins to the mitochondria and peroxisomes (29). In addition, it is currently known that VPA0148 also interacts with TonB-dependent siderophore enterobactin receptor peuA (Fig. 3G) (30). Iron carrier is one of the ways for microorganisms to obtain iron, which gives bacteria more robust adaptability and competitiveness (31). The above analysis shows that these seven TCS genes have the potential in studying *V. parahaemolyticus* regulating macrophages.

### RNA-seq of THP-1 cell-derived macrophages after infection

**Fig 4:**
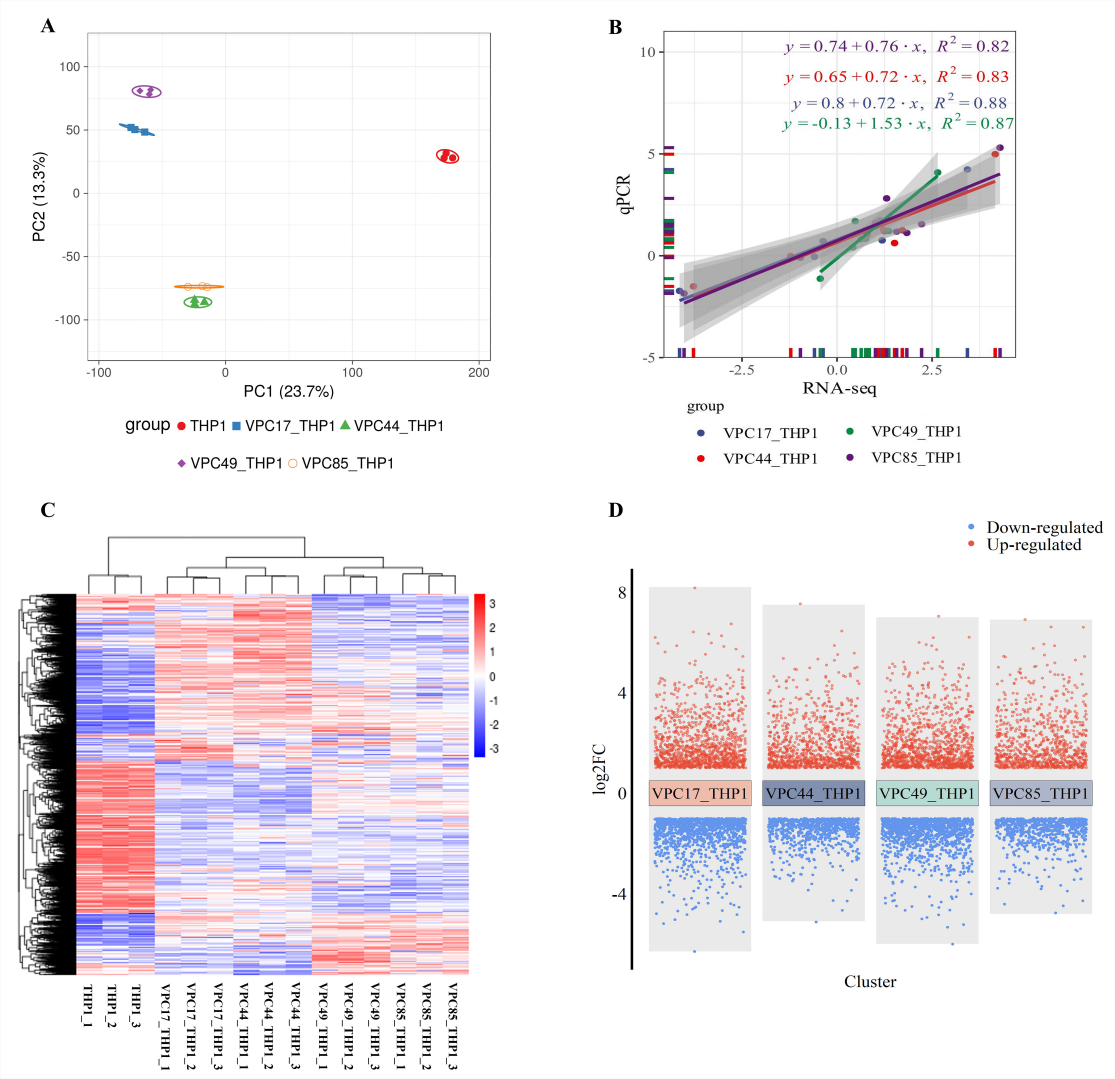
RNA-seq of THP1-derived macrophages after infection. (**A**) PCA of transcript data samples. The transcript data samples were from macrophages. The macrophages were infected with *V. parahaemolyticus* for 1.5 h and treated with the gentamicin-containing medium for 2 h. (**B**) Line regression plot of qPCR and RNA-seq data. (**C**) Cluster heatmap of significant genes (*P*_adj_ < 0.05) in four transcript data samples. (**D**) Multiple volcano plots. The DEG screening conditions were |log2FC| ≥1 and *P*_adj_ < 0.05.

To fully understand the regulation of *V. parahaemolyticus* regulating macrophages at the early stage of infection, RNA-seq was performed on host cells infected with VPC17, VPC44, VPC49, and VPC85 at 3.5 h. Principal component analysis (PCA) analysis of the four groups was shown in Fig. 4A. The sequencing data of VPC17_THP1, VPC44_THP1, VPC49_THP1, and VPC85_THP1 were compared with the reference genome by 96.63%, 96.74%, 96.97%, and 96.93%, respectively. We selected eight genes from each group of transcription samples, a total of 32 genes for qPCR verification. The correlation between qPCR and RNA-seq showed that the expression trends of the two groups were consistent (R^2^ >0.8), and the transcriptome data were credible (Fig. 4B). The sequencing data of uninfected THP-1 cells were used as the control group for downstream analysis. The cluster heatmap of significant genes (*P*_adj_ *<0.05*) revealed that VPC17_THP1, VPC44_THP1, VPC49_THP1, and VPC85_THP1 had similar expression patterns (Fig. 4C). The screening conditions for differentially expressed genes (DEGs) were |log_2_FC| ≥1 and *P*_adj_ < 0.05; the volcanic map showed that there were 2,410, 1,715, 2,369, and 1,576 DEGs in VPC17_THP1, VPC44_THP1, VPC49_THP1, and VPC85_THP1, respectively (Fig. 4D).

**Fig 5:**
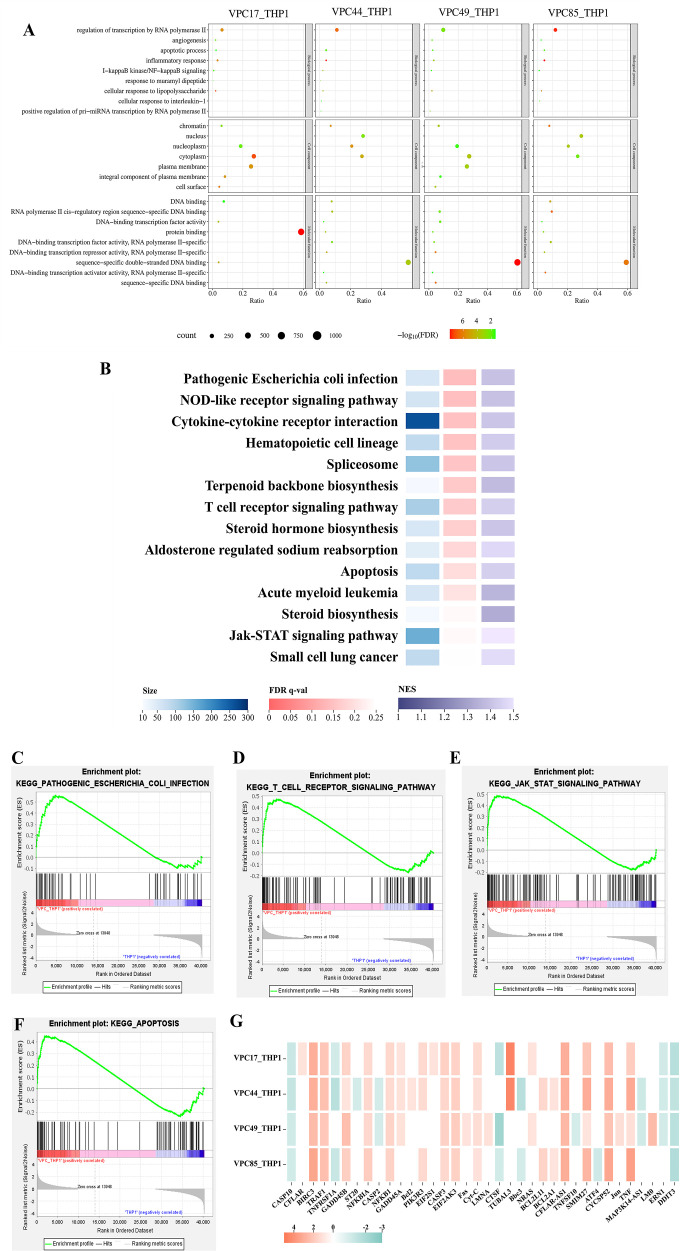
Enrichment analysis of RNA-seq. (**A**) GO enrichment analysis of DEGs in four groups. (B–F) Gene set enrichment analyses of VPC_THP1. The transcriptome data of four groups were summarized as the treatment group, named VPC_THP1, and the uninfected macrophages’ transcriptome data were used as the control group. (**G**) The DEG expression profile of apoptotic pathways in VPC17_THP1, VPC44_THP1, VPC49_THP1, and VPC85_THP1.

Gene ontology (GO) enrichment analysis of DEGs showed that the four groups had similar enrichment results in biological processes, cell components, and molecular functions (false discovery rate [FDR] <0.05) (Fig. 5A). The same biological processes were regulation of transcription by RNA polymerase II, inflammatory response, positive regulation of I-κB kinase/NF-κB signaling, cellular response to lipopolysaccharide, etc. The same cell components were chromatin, nucleoplasm, cytoplasm, and the same molecular functions were DNA-binding transcription factor activity, protein binding, and sequence-specific double-stranded DNA binding. The macrophages exhibited similar immunological responses even when infected with *V. parahaemolyticus* of different genotypes, suggesting that the four strains may share a similar regulatory mechanism. This might be one of the reasons why they were clinically pathogenic, even if they carry different virulence genes. The results indicated that the shared metabolic pathways or DEGs might be the key to *V. parahaemolyticus* regulating macrophages.

Therefore, we used gene set enrichment analysis (GSEA) to evaluate the complete transcriptome of four groups. The transcriptome data of four groups were summarized as the treatment group, named VPC_THP1, and the uninfected macrophages’ transcriptome data were used as the control group. The GSEA results revealed that among the 178 gene sets of VPC_THP1, 87 gene sets were up-regulated, and 14 gene sets were considerably enriched (FDR <25%). The pathways were listed according to FDR (Fig. 5B), including pathogenic *Escherichia coli* infection, NOD-like receptor signaling pathway, cytokine-cytokine receptor interaction, T cell receptor signaling pathway, apoptosis, and other pathways. These pathways pertain to immunological reaction, apoptosis, immune evasion, etc. We pay more attention to the pathways and particular regulated genes associated with immune evasion.

In the pathogenic *Escherichia coli* infection (Fig. 5C) and T cell receptor signaling pathway (Fig. 5D), we found that Cdc42 was up-regulated in all four groups. Cdc42 can affect alterations in cell structure by regulating the actin cytoskeleton that facilitates pathogen invasion or impedes cell function (8). This result indicates that *V. parahaemolyticus* may modify the cytoskeleton actin of macrophages during infection.

In the Jak-STAT signaling pathway (Fig. 5E), interleukin 10 (IL-10) was up-regulated in VPC17_THP1, VPC44_THP1, VPC49_THP1, and VPC85_THP1, respectively (*P* < 0.05). The highly expressed IL-10 can inhibit the inflammatory response and dendritic macrophage activity, lowering the body’s capacity to fight infections and generate immune responses (32). *V. parahaemolyticus* might inhibit the inflammatory response and macrophage activity by stimulating the expression of the inflammatory factor IL-10.

Moreover, all four strains induced apoptosis pathway activation of macrophage (Fig. 5F). We summarized the DEG expression patterns of four groups in the apoptotic pathway (|log_2_FC| ≥ 1 and *P*_adj_ < 0.05) (Fig. 5G). The red square represents up-regulated genes, and the green square represents down-regulated genes. The three classical pathways mediating apoptosis are the mitochondrial, endoplasmic reticulum, and death receptor pathways (33). Although tumor necrosis factor-alpha (TNF-α) was triggered in the death receptor and endoplasmic reticulum pathways, caspase-8 (CASP8) was not activated (Fig. 5G). Meanwhile, caspase-10 (CASP10) was inhibited, and endoplasmic reticulum stress-mediated caspase-12 (CASP12) was not activated (Fig. 5G). In the mitochondrial pathway, the expression levels of Cyt-C and caspase-3 (CASP3) were noticeably up-regulated in four groups (Fig. 5G). Cyt-C can be used as a carrier to transfer electrons in the mitochondrial respiratory chain and establish a transmembrane potential. When it is released into the cytoplasm, it can bind to apoptotic protease activating factor-1 to form an apoptosome and then activate CASP3 to initiate a series of apoptotic reactions (34). The findings implied that *V. parahaemolyticus* might activate the macrophage apoptosis pathway through Cyt-C-mediated mitochondrial pathway.

### The effect of TCS peuS/R in *V. parahaemolyticus*

**Fig 6:**
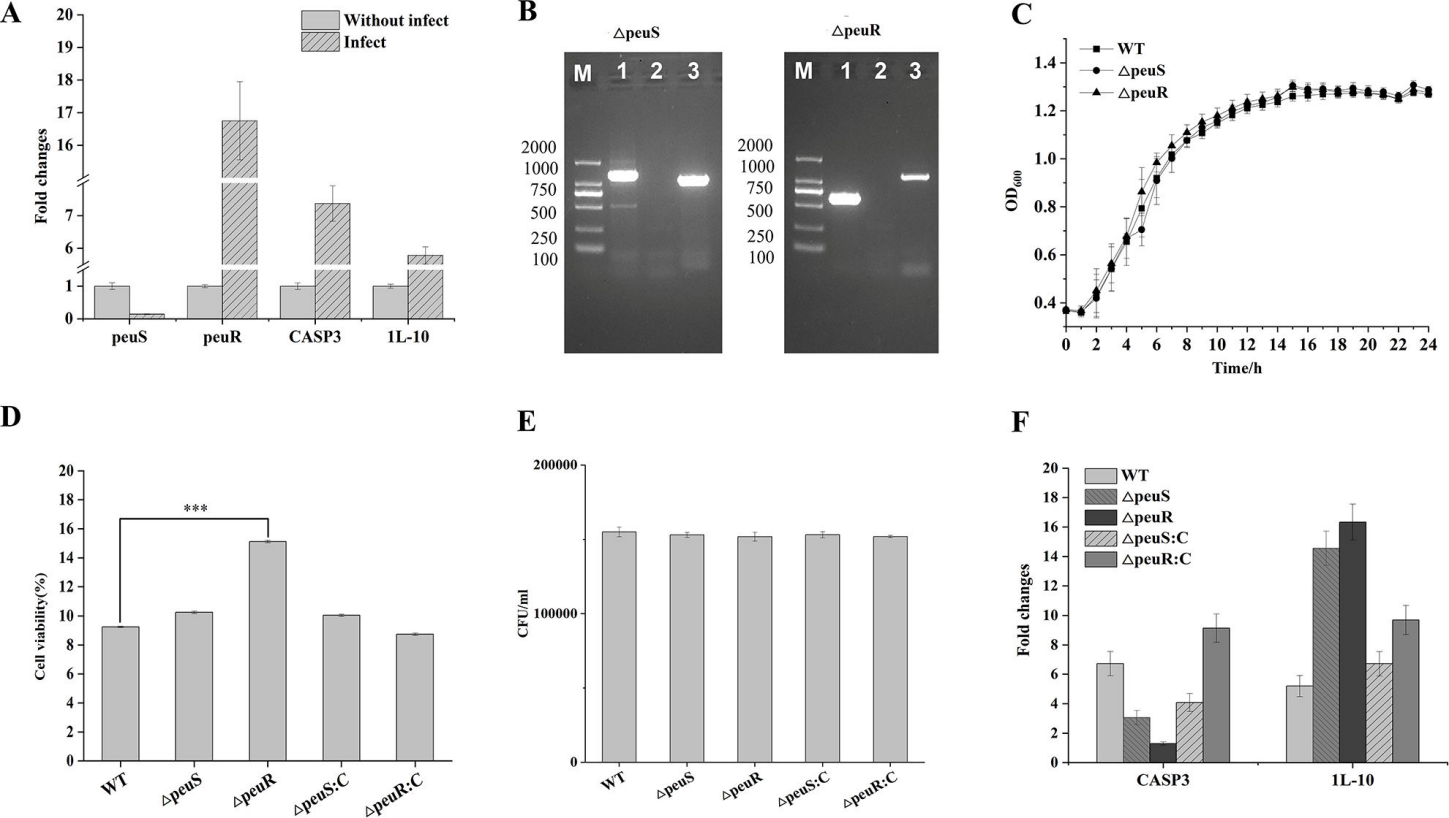
The effect of TCS peuS/R in *V. parahaemolyticus*. (**A**) The expression level of peuS/R in ATCC17802 (WT）after infection and the expression levels of CASP3 and 1L-10 in macrophage after infection. (**B**) Swimming lane 1: peuS/R of WT; swimming lane 2: △peuS/R of WT; swimming lane 3: peuS/R up and down of △peuS/R (note: the target fragment was amplified by peuS/R-up-F and peuS/R-down-R). (**C**) Growth curve of WT and △peuS/R. (**D**) Cell viability of macrophages infected with WT, △peuS/R, and △peuS/R: C. The macrophages were infected with WT, △peuS/R, and △peuS/R: C for 1.5 h and treated with the gentamicin-containing medium for 2 h. (**E**) The number of intracellular WT, △peuS/R, and △peuS/R: C after infection. (**F**) The expression level of CASP3 and 1 L-10 in macrophages after infection with WT, △peuS/R, and △peuS/R: C.

To preliminarily explore the potential role of TCS in *V. parahaemolyticus* regulating macrophages, we selected VPA0148 to construct a mutant strain to infect macrophages. VPA0148 was up-regulated in all four strains, and Tanabe et al. named VPA0149/VPA0148 TCS as peuS/R (30). We selected the model strain ATCC17802 as representative wild-type (WT) to construct peuS/R mutant and complement strains. Firstly, we used WT to infect macrophages for 1.5 h and treated with gentamicin for 2 h. The gene expression trends of peuS and peuR verified by qPCR were consistent with the four strains in this study (Fig. 6A). The expression levels of IL-10 and CASP3 in macrophages were up-regulated by 5.78- and 7.37-folds, respectively (*P* < 0.05). Subsequently, the mutants of peuR and peuS were successfully constructed by homologous recombination (Fig. 6B), named △peuR and △peuS. The growth curve of the △peuR and △peuS showed that the deletion of peuR and peuS did not affect the growth of the strain (Fig. 6C), indicating that peuS/R was not involved in the growth of *V. parahaemolyticus*. Based on the mutant, the complementary strains were successfully constructed and named △peuR: C and △peuS: C, respectively.

The results of the CCK8 assay showed that the survival rate of macrophages infected with △peuR was higher than that of WT (Fig. 6D), indicating that peuR had an inhibitory effect on macrophage growth. The number of intracellular bacteria demonstrated that peuS/R did not impact bacterial invasion and proliferation (Fig. 6E).

After macrophages were infected with mutant and complement strains, the expression level of CASP3 in △peuR_THP1 was significantly lower than that in WT_THP1. When peuR was restored, the gene expression of CASP3 was restored significantly up-regulated compared with WT_THP1, suggesting that peuR may assist *V. parahaemolyticus* in inducing macrophage apoptosis. In addition, △peuS_THP1 also affected the expression level of CASP3, but the one in △peuS: C_THP1 did not recover significantly, suggesting that there may be other HKs that can activate peuR. The expression level of IL-10 in △peuS_THP1 and △peuR_THP1 was significantly higher than that in WT_THP1, indicating that peuS/R may inhibit the expression of IL-10 (Fig. 6F).

## DISCUSSION

In this study, we found that *V. parahaemolyticus* can grow and proliferate in macrophages at the early stage of infection. Bacteria are forced to adapt to huge environmental changes after being taken up by macrophages as foreign bodies. *V. parahaemolyticus* should quickly give response strategies to maintain its survival, such as activating the corresponding signal transduction system and regulating the expression and transcription of related genes. According to the analysis of expression profiles of *V. parahaemolyticus* TCS, the majority of the TCS genes were inhibited or not expressed, and only a small number of genes were up-regulated. The significant change of the TCS genes after infection may result from its interaction with macrophages. We investigated seven crucial TCS genes that might assist *V. parahaemolyticus* control cells, and *V. parahaemolyticus* can change the cell’s actin cytoskeleton, induce inflammatory cytokines, and activate the apoptosis pathway during the infection of macrophages.

Interestingly, the Cdc42 was significantly up-regulated in macrophages infected with *V. parahaemolyticus*. This result resembled Orth’s report that Cdc42 was activated when *V. parahaemolyticus* entered non-phagocytic cells. Moreover, the study showed that *V. parahaemolyticus* activates Rac-GTP and CDC42 through the effector protein VopC, which changes the actin cytoskeleton and promotes bacteria to enter non-phagocytic cells (8). However, VPC17 and VPC44 did not contain VopC (5). But some studies have shown that *V. parahaemolyticus* effector protein, VopS, can mediate the inactivation of Cdc42 in mouse bone marrow-derived macrophages. In this paper, all four strains of *V. parahaemolyticus* had VopS (7), but the relationship between VopS and Cdc42 in THP-1 cell-derived macrophages remains to be further studied.

In this study, *V. parahaemolyticus* may induce macrophage apoptosis through Cyt-C-mediated mitochondrial pathway at the early stage of infection (3.5 h). Apoptosis has two sides to bacterial infection. On the one hand, apoptosis is one of the means by which host cells eliminate bacteria. On the other hand, the induction of apoptosis cannot always protect host cells in infection because bacteria can use the host’s apoptosis mechanism to eliminate the cells required for immune response (35). As a result, apoptosis serves a variety of intricate roles in bacterial infection regulation. However, combined with the amounts of intracellular bacteria (Fig. 1B), we discovered that *V. parahaemolyticus* was still undergoing multiplication at 3.5 h. The number of intracellular bacteria started to decline after 4.5 h. We speculated that *V. parahaemolyticus* might trigger apoptosis to aggravate cell rupture, which helps *V. parahaemolyticus* detach from cells.

To investigate the potential role of *V. parahaemolyticus* TCS in the regulation process, we infected macrophages after knocking out the TCS genes HK (peuS) and RR (peuR). The results revealed that △peuR had lesser toxicity than WT, suggesting that peuR had some involvement in the process of infection. In the TCS, the peuS is HK which detects external signals and phosphorylates the downstream peuR. And the peuR is an RR that primarily controls the transcription of particular downstream genes. However, the results suggested that there may be other unknown genes that can activate peuR. TCS is a complex regulatory mode with classical HK and RR modes but also has cross-regulation modes, which was also reflected in the PPI of peuR. Our research indicated that TCS might be involved in the *V. parahaemolyticus* infecting macrophages.

### Conclusion

In summary, our work understood the interaction between *V. parahaemolyticus* and macrophages from two aspects of bacteria and cells. From the perspective of bacteria, the expression patterns of TCS in *V. parahaemolyticus* with different genotypes infected macrophages were characterized and the multiple vital genes for *V. parahaemolyticus* immune escape were provided. From the perspective of cells, the analysis of the cell gene alterations has revealed that *V. parahaemolyticus* might infect macrophages by controlling apoptosis, actin cytoskeleton, and cytokines. In addition, we also identified that peuS/R in TCS might affect the toxicity of *V. parahaemolyticus* to macrophages and might play a role in the induction of apoptosis, which supported the study of *V. parahaemolyticus* TCS in innate immunity regulation. This study provided several new insights into the pathogenicity of *V. parahaemolyticus*.

## MATERIALS AND METHODS

### Bacterial strain and cells culture

Four *V. parahaemolyticus* strains were stored in our laboratory (Table 2) (5). The tryptic soy broth with 3% NaCl (wt/vol) (TSB + N) was used for culture bacteria. The THP-1 cell line was maintained in RMID1640 (Gibco, New Zealand) supplemented with 5% fetal bovine serum (FBS) (Gibco, New Zealand) in a humidified 5% carbon dioxide atmosphere. All strains and cells were cultured at 37℃.

**TABLE 2:** Four strains of *V. parahaemolyticus^a^*

Strain name	*trh*	*tdh*	toxR	Source
VPC17	−	+	+	Fecal specimens with acute diarrhea
VPC44	−	−	+	Fecal specimens with acute diarrhea
VPC49	+	+	+	Fecal specimens with acute diarrhea
VPC85	+	−	+	Fecal specimens with acute diarrhea

^*a*^ "+" means positive; "−" means negative.

### Infection and intracellular survival bacterial counts

The THP-1 were seeded at 2 × 10^5^ cells in 12-well plates , and added phorbol 12-myristate 13-acetate (PMA) (Sigma, USA) with a final concentration of 100 nmol/L to induce cell transformation into macrophages for 48–72 h. Overnight-grown bacteria were induced in TSB + N medium containing 0.05% (wt/vol) bile salt for 2 h and diluted with RMID1640. The cells were infected with bacteria at a multiplicity of infection (MOI) of ~10 per cell and then centrifuged at 200 × *g* for 5 min to synchronize the infection (17). At the specified times, the culture medium was removed, rinsed with 1× phosphate buffered saline (PBS), and treated with an infection media containing 100 ng/mL gentamicin. The number of intracellular bacteria is counted by CFU/mL at the indicated time points. Host cells were washed with 1× PBS to remove extracellular dead bacteria and lysed with 1% (vol/vol) TX-100 (8).

### qRT-PCR

The gene expression level was identified by qRT-PCR (Roche Light Cycler 480, USA). RNA extraction and cDNA transcription were performed according to the kit steps (Vazyme, Nanjing, China). And the primers for TCS genes were shown in Table 3. The housekeeping gene of *V. parahaemolyticus* was 16S rRNA. The fold changes of relative gene expression were determined by the 2^–ΔΔCT^ method (7).

**TABLE 3:** Primer sequences of TCS genes

Gene name	Primer	Primer sequences (5´−3´）
16S-rRNA	16S-rRNA-F	GCCTTCGGGAACTCTGAGACAG
	16S-rRNA-R	GCTCGTTGCGGGACTTAACCCAA
glnL	glnL-F	AGCGCTTTGCGAAAACAACA
	glnL-R	ACCAGTCTCCCTAGTGCCAT
envZ	envZ-F	GGTGAAAATCAGCACAGGCG
	envZ-R	CCAGACCAGTACCTTCGCTG
VP_RS01745	VP_RS01745-F	CGCCATTGAACGCCACTTTT
	VP_RS01745-R	CAGCTTGCCTTCAAACACCC
arcB	arcB-F	GCCCATGAAAAATCTCGCCC
	arcB-R	ATATCATCGACGCGCCCTTT
VP_RS02565	VP_RS02565-F	TGGTCGAAGGGTTTGGCTAC
	VP_RS02565-R	GGCTTTACCGACCTAGCCTG
phoR	phoR-F	AGAACTGCGTATCGTGCCTT
	phoR-R	CATCGGAGTGCGTAGCTCAT
VP_RS04455	VP_RS04455-F	TTTACGACCACGATGCCCAA
	VP_RS04455-R	TACGCTCTGGGCTCAACATC
VP_RS05205	VP_RS05205-F	GAAGTTTACTGACAGAGGCA
	VP_RS05205-R	TATCCCTATTCCCGTGTCTT
VP_RS05835	VP_RS05835-F	GGACGCCAATCGAGAAAAGC
	VP_RS05835-R	ACCTCGCCCGAATACATCAC
VP_RS05880	VP_RS05880-F	ATAGCATCCACTCGCTCTGC
	VP_RS05880-R	GCTAGAGCCCAAAACGCAAG
VP_RS06040	VP_RS06040-F	CAAAGCATACGCGGAAGTGG
	VP_RS06040-R	GCTGACATGGTGAAGAGCCT
VP_RS06690	VP_RS06690-F	TTAGTGTGCGTTTCAGCCCT
	VP_RS06690-R	CGCATCGCACGCATTCTTTA
VP_RS07285	VP_RS07285-F	TACGCGAGTGAGTGCAGAAG
	VP_RS07285-R	GCGATGTTGGTCATGTACGC
VP_RS07485	VP_RS07485-F	CGTTACAACCAAAGCCGTCC
	VP_RS07485-R	GAAATGTAAGCGCCTCTGCG
VP_RS08245	VP_RS08245-F	TGTCGAGCAAATACCCCACC
	VP_RS08245-R	TCTACAGCGCGTGGTTCTTT
VP_RS08360	VP_RS08360-F	GCCCGGAGATTCCACAAGAA
	VP_RS08360-R	TGTAGCGTGGCGATGTCTTT
VP_RS08440	VP_RS08440-F	ACCGTCAGCATTAGCCCAAA
	VP_RS08440-R	CTCTACACTGCCTCGTTCCG
VP_RS09135	VP_RS09135-F	GTTCAAGGCTGAGGTGACGA
	VP_RS09135-R	CCTATCGCTCGAACACCCAA
VP_RS09270	VP_RS09270-F	TCCGTGCCTAAACCAAGCTC
	VP_RS09270-R	AGACGATGGCGTAGGGTTTG
VP_RS09570	VP_RS09570-F	TCAGAGGGCGGCATTTTCAT
	VP_RS09570-R	AACCGTCAGCGCAACAAAAA
VP_RS09640	VP_RS09640-F	GTCAAGAGCCGAACACCAGA
	VP_RS09640-R	CACCGCCAGTACCATTACGA
VP_RS09770	VP_RS09770-F	TCTTCATGCGGACGCTTCTT
	VP_RS09770-R	GCGATTGTCGGGGAAATTGG
cheA	cheA-F	TGGCACTTCGTTAGCAGGTT
	cheA-R	CTGGATGAACTGCACCGTCT
VP_RS10935	VP_RS10935-F	CAGAAATCGCCAAACGCACT
	VP_RS10935-R	CGACACGTATGGTGTTGTGC
VP_RS12030	VP_RS12030-F	ATCGGTCGCTTGGCTATCAG
	VP_RS12030-R	GCAATTCGTGCGAGGTTGTT
barA	barA-F	GTTGATCGCATTCGTCGTGG
	barA-R	CCAAGGTTTCGCGTAGGTCT
cpxA	cpxA-F	ACGCATTTTATTCCGCGTCG
	cpxA-R	CAGTTGAAAGTTGCTGCGCT
VP_RS14135	VP_RS14135-F	CACCGCAATCGAGAGGCTAT
	VP_RS14135-R	GTGGTTCCCGTTTCTTTGGC
VP_RS15315	VP_RS15315-F	TTGCTGTTGATGGTTGGGGT
	VP_RS15315-R	ACCGCCGCTAAAATCACTCA
VP_RS15360	VP_RS15360-F	GCGCTGTTTTTCTCGTGGTT
	VP_RS15360-R	AACAACAAACACGCGGTCAG
VP_RS16000	VP_RS16000-F	TGAAGCTGGTGTGTCCATCC
	VP_RS16000-R	GCTGATGACTATCGCGGTCA
VP_RS16155	VP_RS16155-F	TGAGATTGATAGACACGCAG
	VP_RS16155-R	CTCAAAACATTCGCGTACAA
torS	torS-F	CGAAACGAGCGACACCAATC
	torS-R	CCAAGTACATCCGCACTGGT
VP_RS18590	VP_RS18590-F	ACGCATTTCATCATCGCAGC
	VP_RS18590-R	CTGAGCCATTGTCCGTCACT
VP_RS18720	VP_RS18720-F	TTGTTGCCTTGTCGTTGGGA
	VP_RS18720-R	CTGCAATAAACGCCACAAAGT
VP_RS18835	VP_RS18835-F	CGGCCTGTGAGTACACCATT
	VP_RS18835-R	AAGCCCAATTCGCACAACAC
VP_RS19135	VP_RS19135-F	ACTGAACAAGCCATGCAGGT
	VP_RS19135-R	TTTCCAGTAGCGAGGCAAGG
VP_RS19580	VP_RS19580-F	TTGACGCAATCGGCGAGTAT
	VP_RS19580-R	CGTAGCCGTATGCTGGTCTT
uhpB	uhpB-F	TCTGCTTTTCCCATTCGCCT
	uhpB-R	CGTCAGCGCCCATTCTGATA
VP_RS20325	VP_RS20325-F	GTGCTTATCCTCTTCGCCCA
	VP_RS20325-R	AAGGCCCCGTTACCAAACAA
VP_RS20450	VP_RS20450-F	GAGAAATCCCGAGCACACCA
	VP_RS20450-R	AAGCCGAGACTGACAAAGCA
VP_RS20585	VP_RS20585-F	AACGTGATTGCTTTCGCCAC
	VP_RS20585-R	TTGCACGCTCTGTCGTAAGT
narQ	narQ-F	CGATTAAAGGTGCGCGTGAG
	narQ-R	TCCGTTTGGAAGTCGTGAGG
VP_RS21020	VP_RS21020-F	CTTCTGCTCTTCCGCTTCCA
	VP_RS21020-R	GCGAGCTGACTTCTTGGCTA
VP_RS21060	VP_RS21060-F	CCATACCAAGCATCGCCTCT
	VP_RS21060-R	GACGCCAATACGGTCAGGAA
VP_RS21285	VP_RS21285-F	TGGAACGCCCAGAAACAGAG
	VP_RS21285-R	GCGTCTCTTGCTGCCATTTG
VP_RS22020	VP_RS22020-F	AATCTCTCCTTGGTGTGCGG
	VP_RS22020-R	TGTTCGGGACATGGGGATTG
VP_RS22410	VP_RS22410-F	GACGAGAACTCCCGATCACC
	VP_RS22410-R	CCCGATTCTAGGCGATGACC
VP_RS23125	VP_RS23125-F	TCGCGGTCTTTAATGTGGCT
	VP_RS23125-R	TGTCGAGCCGATTAGACGTG
VP_RS23425	VP_RS23425-F	ACGCTCTAACAAACGGGGTT
	VP_RS23425-R	TTTTACCGTGATGACGCCGA
glnG	glnG-F	TAGCGGCATCTTGCTTGTGA
	glnG-R	GCTGCGGGGGATAAAGAACT
ompR	ompR-F	CATCGGGTGAATTTGCGGTG
	ompR-R	ATGCGGCGTAGACGAGAAAT
VP_RS01740	VP_RS01740-F	AAACTCGATGGTTGGCAGGT
	VP_RS01740-R	GCGATCTTCCACTTGCTCCT
arcA	arcA-F	CTCTTCACCGTCACTGGCTT
	arcA-R	ACCCCGCAGATTCTTATCGT
btsR	btsR-F	GATGGCATTGGACGCATCAC
	btsR-R	CTCGAGAAGAGCTGGCAGAG
phoB	phoB-F	TCGCGGTTTAGAGGTTGGTG
	phoB-R	GCGTAACACGGCGAATAACC
VP_RS03810	VP_RS03810-F	GACGGGGATTCTTGACTCGG
	VP_RS03810-R	CCCCATCAGTCGAAAGGTCA
VP_RS04450	VP_RS04450-F	ATGGTTTCTCGCAGCGTTTG
	VP_RS04450-R	TTTTGCGCCAGAACACTTGG
torR	torR-F	GGAGCAAGTAAAGCGGCAAG
	torR-R	ACGTTCACGACTCAGCACTT
VP_RS05840	VP_RS05840-F	CGAATGCCAGAAATGCGTGG
	VP_RS05840-R	AAGCATTACCGTCCACAGGC
VP_RS05885	VP_RS05885-F	TAGGATCACGACCGGAGTGT
	VP_RS05885-R	AGCGACATCCGAAGAATGGG
VP_RS06035	VP_RS06035-F	AGCTGATGCCCGTTTGTTCT
	VP_RS06035-R	ATCGTTGAGGGTGCAGAGTC
VP_RS06695	VP_RS06695-F	GATGCCAGGTAAAACGGGTG
	VP_RS06695-R	ACAAGACTTTGCGAGTGCGA
VP_RS07125	VP_RS07125-F	CTTCACGTTCGACTTGCCAC
	VP_RS07125-R	AGCCCGAGCAACAATCTCAA
VP_RS07185	VP_RS07185-F	TGGCGAAAGGTTGGGGTATC
	VP_RS07185-R	CCGGCTGACGAAGTGAAAAC
VP_RS07280	VP_RS07280-F	CGAGGCATTCACCAAGCAAG
	VP_RS07280-R	GCTACGCGGAACAAACCTTC
VP_RS08240	VP_RS08240-F	GCGGCTTCACCAACAAACAA
	VP_RS08240-R	TTTGGTTGACCGCATGTTGC
VP_RS08355	VP_RS08355-F	TGGGTGGAAAAAGGGTCTGG
	VP_RS08355-R	GAATGTCGTCTCGTGCGGTA
VP_RS09265	VP_RS09265-F	CTCAAGACCGCCTCTTCTCG
	VP_RS09265-R	TGGTGCAAGCCATCAAAACC
uvrY	uvrY-F	GGGTTTTCAGAGGCTGGTGA
	uvrY-R	TCGTGTGGTCAATAGTGGGC
VP_RS09635	VP_RS09635-F	GCGGGTAATAACACTCCGGT
	VP_RS09635-R	CGGTTTGGTGAGGTAGTCGT
VP_RS09765	VP_RS09765-F	AATTCTGCGACGGTTTTGGC
	VP_RS09765-R	CGAATTGCGGATGAGCTGTG
VP_RS09900	VP_RS09900-F	CTACCGCTGAGTGACGTGTT
	VP_RS09900-R	GCCGAGATGGTTTAGAGGCA
VP_RS10210	VP_RS10210-F	CGTTACCCGGCCACTCATAA
	VP_RS10210-R	TTCCTTTGCATCTTCCGCCT
VP_RS10600	VP_RS10600-F	GCTCTCACCAGTAGCGTTGT
	VP_RS10600-R	CTCTCAAGGTGACTGGCGTT
VP_RS10815	VP_RS10815-F	ATCCATGGTGATGACGTCC
	VP_RS10815-R	CGTTGCGGTAAATGGCAGAG
cheY	cheY-F	CGATACCTTGCATTCCTGGC
	cheY-R	ATTTCTCAACAATGCGCCGT
VP_RS10930	VP_RS10930-F	TAACGCGGTTGACGGATTCA
	VP_RS10930-R	TGCTAGAAGGCGTGGATTGG
VP_RS14060	VP_RS14060-F	CAGTGAAGCAAACGACGGTG
	VP_RS14060-R	GGTTTCCCAGTTCTCTCGCA
VP_RS14100	VP_RS14100-F	AGGAAGAGCCGGATTTGGTG
	VP_RS14100-R	AAAGCCAAACGCACCATGAC
VP_RS15365	VP_RS15365-F	CGCCTCAAACACCGCAATAC
	VP_RS15365-R	TCTTTGAGCGATGTGCGGAT
VP_RS15995	VP_RS15995-F	TTGCTCGATGTGATGTTGCC
	VP_RS15995-R	CGCATTAGCAACTCAGCGAC
VP_RS16160	VP_RS16160-F	TATCCAAGCGCCCGTTATCC
	VP_RS16160-R	CGTCTGCGCTCGATAAGTCT
VP_RS17300	VP_RS17300-F	ACGTTCACGCTCTACATCCG
	VP_RS17300-R	ATTGAAACGCCACCAGCAAG
VP_RS18725	VP_RS18725-F	GACACGTTCCAAACCAGTGC
	VP_RS18725-R	AGCCAGTGCCAAAGTCATCA
VP_RS18730	VP_RS18730-F	GCTGCAAGGCAAGTCCAATC
	VP_RS18730-R	CCGCTACTCTCGTTAGCTCG
VP_RS18745	VP_RS18745-F	CATCGAGTAACCGCGAGACA
	VP_RS18745-R	TTTAGCGACGGGCTATCGTG
VP_RS18750	VP_RS18750-F	ATTCTTGCAGGCGCATTTCC
	VP_RS18750-R	AGCGGTACTAGCCAGCAATC
VP_RS18760	VP_RS18760-F	TCGTTCGCAAAGATGGCAAC
	VP_RS18760-R	AGAACGGATGAACGCGGAAT
VP_RS18840	VP_RS18840-F	CGTCAGGTGGTGGATCTGTT
	VP_RS18840-R	GCCCTACTTTCCCGTGTTCT
VP_RS19140	VP_RS19140-F	CCTGAACTGCCTGAAGAGGG
	VP_RS19140-R	GCAATGGCTGTACGTGAACC
VP_RS19575	VP_RS19575-F	AGGTAGCTGGCGATCTAGGA
	VP_RS19575-R	CTGCTTCTCGTTGAGGACGA
VP_RS19615	VP_RS19615-F	CTTGAATGCGCTCTGCTTCC
	VP_RS19615-R	GTTGGTCGTTATGGCGGAGA
uhpA	uhpA-F	GCTGTAGCCCAGACGAACTT
	uhpA-R	GCGATGCAGCTTTGTCACTT
VP_RS20190	VP_RS20190-F	TGCTGGCTTCACAACCTCTC
	VP_RS20190-R	AAAAACGCGGTCGCACATAC
VP_RS20210	VP_RS20210-F	AACGTAATTTCCCCGCCCTT
	VP_RS20210-R	ACGCAAACAGGCAAACTGAG
VP_RS20330	VP_RS20330-F	TGGACCACGACTAGCCAAAG
	VP_RS20330-R	CGGGACGCCCATTCTTTTTC
VP_RS20735	VP_RS20735-F	AGCTCAATCACGCTTGGTGT
	VP_RS20735-R	CAACCCAAAGCCAAAGAGCG
VP_RS20890	VP_RS20890-F	CCTGATGGGCCTGTTTGAGT
	VP_RS20890-R	GACAGCCCTGCGGATATTGA
VP_RS21280	VP_RS21280-F	CGACGGTGAGTTTGACTTGC
	VP_RS21280-R	GTCGTACTCAATCCCGCGTA
VP_RS22015	VP_RS22015-F	GAACCAGCACTTTGATCCGC
	VP_RS22015-R	AGATTTAGGCTTGCCCGACG
VP_RS22415	VP_RS22415-F	CAGTCAACACCAAAACCGGC
	VP_RS22415-R	GTCAGTCGAGAATCCAGCGT
VP_RS22515	VP_RS22515-F	ATGCGAGGAGAGTGTTCGTG
	VP_RS22515-R	TTGAAGCTCTCGGCAAACCA
VP_RS23130	VP_RS23130-F	GGTTGACGAACAAACCGAGC
	VP_RS23130-R	TCGACCGCCAATAGCAAACT
VP_RS23430	VP_RS23430-F	ACCAAGGTTGATGCCGATGT
	VP_RS23430-R	GTCCAAACTGATGTGCGGTG

### RNA sequencing, GO, and GSEA

After 1.5 h of infection at 37°C, cells were treated for 2 h with infection media containing 100 ng/mL gentamicin, and host cells were washed with 1× PBS in preparation for total RNA extraction. RNA sequencing was performed in Allwegene Gene Technology Co., Ltd. (Nanjing, China), and the platform is Illumina NovaSeq 6000. The genes were identified by qRT-PCR, the primers were shown in Table 4, and the housekeeping gene of the cell was GADPH. The bioinformatics tool Database for Annotation, Visualization, and Integrated Discovery (https://david.ncifcrf.gov) performed GO enrichment analysis on the DEGs (36). GSEA was applied to the whole transcriptome by the GSEA tool, and the KEGG database from MSigDB (v7.4) was used for the GSEA of the cell. The number of random combinations was 1,000. The gene set under the pathway of |NES| >1, NOM *P*-value <0.05, and FDR q-value <0.25 is significant (17, 37).

**TABLE 4:** Primer sequences of genes

Gene name	Primer	Primer sequences (5´−3´）
GAPDH	GAPDH-F	GGAGTCCACTGGCGTCTTCA
	GAPDH-R	GTCATGAGTCCTTCCACGATACC
BCL2	BCL2-F	GGATTGTGGCCTTCTTTGAGTTC
	BCL2-R	CTTCAGAGACAGCCAGGAGAAAT
IL10	IL10-F	CCGTGGAGCAGGTGAAGAATG
	IL10-R	CCAGAGCCCCAGATCCGATT
CASP3	CASP3-F	GGAATGACATCTCGGTCTGGT
	CASP3-R	GCACACAAACAAAACTGCTCC
CDC42	CDC42-F CDC42-R	CCCTCTACTATTGAGAAACTTG AGAACACTCCACATACTTGA
DDIT3	DDIT3-F	TCATGTTAAAGATGAGCGGGTGG
	DDIT3-R	TGCTTTCAGGTGTGGTGATGTAT
PIM1	PIM1-F	TTCGGCTCGGTCTACTCAGG
	PIM1-R	TTAGGCAGCTCTCCCCAGTC
RHOA	RHOA-F RHOA-R	ACTATGTGGCAGATATCGAGGTGGA TATCAGGGCTGTCGATGGAAAAAC`
STAT3	STAT3-F STAT3-R	CAAGCAGTTTCTTCAGAGCA CGTCACCACGGCTGCTGT

### Construction of deletion mutations

The allelic exchange was used to create the deletion mutations using pDS132, which contains the upstream and downstream sequences of the target genes (38). Briefly, using certain primers with restriction enzyme sites, the target gene’s upstream and downstream homology arms were amplified and combined into the suicide plasmid pDS132. After being inserted into *E. coli* S17pir, the recombinant plasmid was conjugated into WT *V. parahaemolyticus*. A sacB counter-selectable marker and a chloromycetin resistance cassette are present on the plasmid, which could exchange genetic fragments twice with the genomes of *V. parahaemolyticus* by intermolecular recombination. PCR was used to identify the potential deletion mutant, and sequencing was used to confirm it. Based on the mutant strains, pBAD33 was used to complement the mutant gene to construct the complemented strain. The primer sequence, strains, and plasmids were shown in Tables 5 and 6.

**TABLE 5:** Strains and plasmids

Strains/plasmids	Description	Source
Strains		
*V. parahaemolyticus*		
ATCC17802	Isolated from “Shirasu” food poisoning in Japan	Our laboratory
△*peuR*	*PeuR-*deleted mutant from ATCC17802	This study
△*peuS*	*PeuS-*deleted mutant from ATCC17802	This study
*E. coli* S17λpir	TpR, SmR, recA, thi, pro, hsdR-, M+, RP4: 2-Tc: Mu: Km Tn7 λpir	Biomedical
Plasmids		
pDS132	Cmr, sucB, a sucrose-suicide plasmid	Biomedical
pDS132: *peuR-*up and down	Cmr, sucB	This study
pDS132: *peuS-*up and down	Cmr, sucB	This study
pBAD33	Cmr, a gene expression vector used for the complementary experiment	Biomedical
pBAD33: *peuR-*up and down	Cmr	This study
pBAD33: *peuS-*up and down	Cmr	This study

**TABLE 6:** The primer sequence of the mutant gene

Use	Name	Sequence (5´–3´)
For deletion	*peuR-*up*-F*	aaaaaggatcgatcctctagaAATCGATGCTGATGAGTTGCTCG
	*peuR-*up*-R*	cacaccagcttcatGCCAAATTACCACTTAAATAAAAATGG
	*peuR-*down*-F*	tttggcATGAAGCTGGTGTGTCCATCCT
	*peuR-*down*-R*	gtggaattcccgggagagctcCATTAAAATCGCCGTTAGAAAGC
	*peuS-*up*-F*	aaaaaggatcgatcctctagaCGATGTGATGTTGCCAAACTTAA
	*peuS-*up*-R*	acttcattgttacccgCACGCGCCCACAGATTCTA
	*peuS-*down*-F*	cgtgCGGGTAACAATGAAGTGAACTCG
	*peuS-*down*-R*	gtggaattcccgggagagctcGCGCTCTAAAAGCTCAATTGGC
For PCR	*peuR* -F	GCATGTCTCGAGTTCTGATTGT
	*peuR* -R	ACGCGCCCACAGATTCTAAA
	*peuS* -F	TGATGAAGCTGGTGTGTCCA
	*peuS* -R	GTCTGAACACCAAACTCACGG

### Cell viability

To determine if the experimental treatment was harmful to cell survival, the Trans Detect Cell Counting Kit-8 (Sangon Biotech, Shanghai, China) was employed. 1 × 10^4^ cells were put into each well of a 96-well plate. The cells were then incubated at 37℃, 5% CO_2_. After that, 10 µL of CCK-8 was added to the wells, and the cells continued to incubate for another hour. Finally, optical density values were calculated at 450 nm wavelength (39).

### The expression of CASP3 and IL-10

RNA was extracted from macrophages infected with WT and mutations. The expression of CASP3 and IL-10 was identified by qPCR, and the primers were shown in Table 4.

### Statistical analysis

PPI networks used String (https://cn.string-db.org/). The visual data graphs were drawn by ClustVis (40), Hiplot (41), and ChiPlot(https://www.chiplot.online/). Multiple volcano plots were drawn by R version 4.2.2. All experiments were performed with at least three biological replicates, and all data are presented as mean ± standard deviation.

## Data Availability

The sequencing data have been submitted to the NCBI BioProject under the accession number PRJNA940201.
